# The Chiral Pool in the Pictet–Spengler Reaction for the Synthesis of β-Carbolines

**DOI:** 10.3390/molecules21060699

**Published:** 2016-05-27

**Authors:** Renato Dalpozzo

**Affiliations:** Dipartimento di Chimica e Tecnologie Chimiche, Università della Calabria, Arcavacata di Rende (Cs) 87030, Italy; renato.dalpozzo@unical.it; Tel.: +39-0984-492055

**Keywords:** Pictet-Spengler reaction, asymmetric synthesis, β-carboline, alkaloids

## Abstract

The Pictet–Spengler reaction (PSR) is the reaction of a β-arylethylamine with an aldehyde or ketone, followed by ring closure to give an aza-heterocycle. When the β-arylethylamine is tryptamine, the product is a β-carboline, a widespread skeleton in natural alkaloids. In the natural occurrence, these compounds are generally enantiopure, thus the asymmetric synthesis of these compounds have been attracting the interest of organic chemists. This review aims to give an overview of the asymmetric PSR, in which the chirality arises from optically pure amines or carbonyl compounds both from natural sources and from asymmetric syntheses to assemble the reaction partners.

## 1. Introduction

**Scheme 1:**
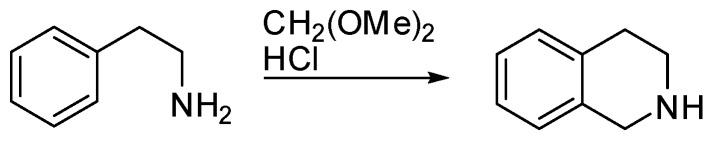
The Pictet–Spengler reaction.

The reaction named the Pictet–Spengler reaction (PSR) was discovered by Amè Pictet and Theodor Spengler in 1911. They heated β-phenylethylamine and formaldehyde dimethylacetal in the presence of hydrochloric acid and recovered 1,2,3,4-tetrahydroisoquinoline as the product (Scheme 1) [1].

For over a century, it has been used for the synthesis of a large variety of aza-heterocyclic compounds. A seminal asymmetric synthesis was already presented in the work of Pictet and Spengler, when they tested phenylalanine and tyrosine in their reaction. Tryptamine as the amine component was introduced 20 years later [2], allowing the creation of the 1,2,3,4-tetrahydro-β-carboline skeleton, and, consequently, the synthesis of many indole alkaloids of enormous physiological and therapeutic significance [3].

After the work of Pictet and Spengler, the first asymmetric PSR was only performed in 1977, when an enzyme of the Pictet–Spenglerase class was discovered [4]. The enzyme-catalyzed PSR’s has been recently reviewed [5].

During the last two decades, the increasing development of the asymmetric synthesis by chiral catalysts or chiral precursors has produced remarkable progress in developing new and highly enantioselective PSRs. This review aims to provide an overview of the asymmetric PSR, in which the chirality arises from optically pure amines or carbonyl compounds both from natural sources and from asymmetric syntheses to assembly the reaction partners. However, external asymmetric induction by chiral catalysts on symmetric derivatives can also be used in PSR [6,7,8,9,10], but these examples will not be covered in this review.

**Scheme 2:**
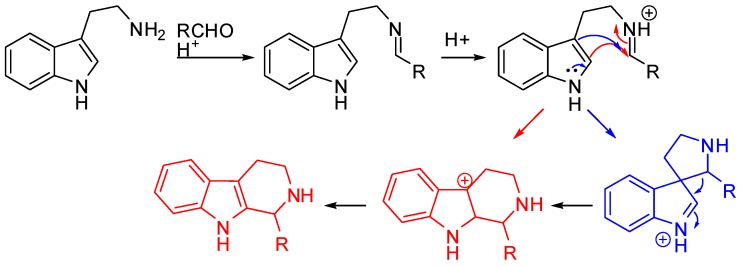
The mechanism of the Pictet–Spengler reaction.

The Pictet–Spengler reaction starts from the formation of an imine, which, under the acidic reaction conditions, is turned into an iminium ion followed by nucleophilic attack by the aryl group and cyclization. In the case of tryptamine, the attack on the iminium ion can occur either directly at position 2 (Scheme 2, red pathway) or at position 3 of the indole to form a spiroindolenine (Scheme 2, blue pathway) [11,12]. However, spiroindolenine undergoes fast rearrangement, and the carbonium ion is the final product anyway [13,14].

## 2. Starting from Tryptophan Derivatives

**Scheme 3:**
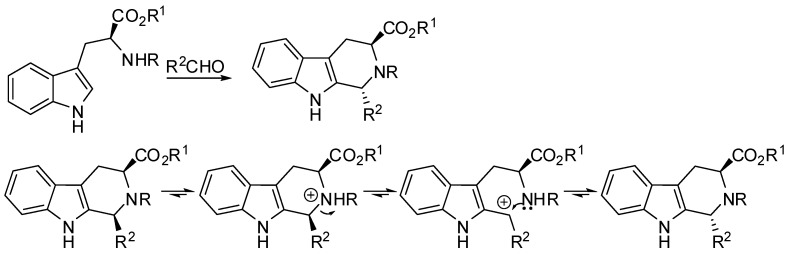
Diastereoselectivity of the PSR of tryptophan derivatives and *cis* to *trans* epimerization in the PSR.

The first natural chiral source used to prepare β-carbolines was tryptophan and its derivatives. In fact, since 1979, Cook and co-workers studied the reaction of these compounds with aldehydes [14,15,16,17,18]. Thus, they found that the steric bulk of the carbonyl compounds, and the substituents either at the N_b_ nitrogen atom or at the ester group governed the diastereoselectivity of the PSR, leading preferentially to *trans*-1,2,3-trisubstituted tetrahydro-β-carbolines under thermodynamic stereocontrol (Scheme 3).

If the reaction mechanism involved the spiroindolenine, the *trans*-isomer of which is more stable, the following ring expansion was therefore stereospecific. When a mixture of isomers was obtained, the thermodynamically more stable *trans* isomer could be exclusively obtained by epimerization of the *cis* isomer under acidic conditions [19,20,21]. Between the two possible mechanisms of the epimerization process, the protonation of the N_b_, followed by ring-opening and closure to give the more stable isomer was demonstrated to occur (Scheme 3). There was no evidence of an alternative protonation of the indole-nitrogen (N_a_) with an olefinic isomerization. However, under these conditions the racemization of the products was possible [22]. By this methodology, besides the application of Cook’s group to the preparation of natural products [18,23,24,25,26,27,28], a new class of inhibitors of *Mycobacterium tuberculosis* protein tyrosine phosphatase B was synthesized by Waldmann and co-workers [29].

**Scheme 4:**
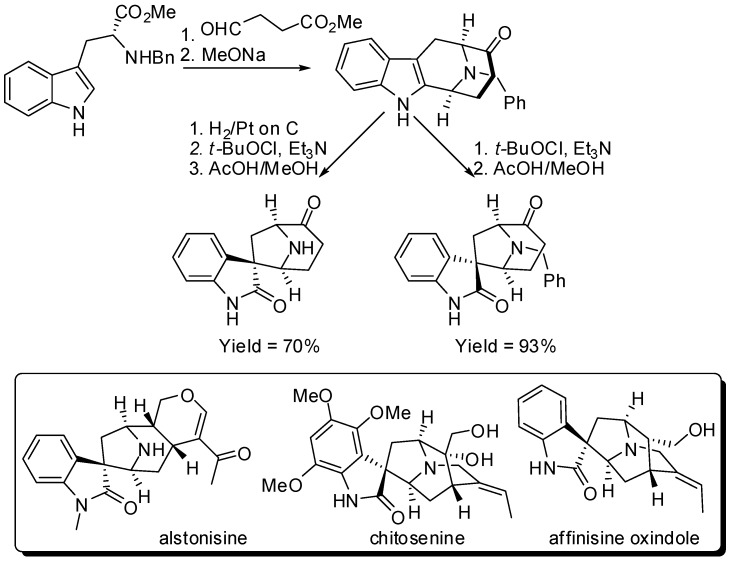
Synthesis of the chitosenine, alstonisine and affinisine skeletons.

The total synthesis of (−)-affinisine oxindole was also completed in an enantiospecific fashion from d-tryptophan in 10% overall yield. The key step was an asymmetric Pictet–Spengler reaction carried out under Cook’s conditions on a 300-gram scale [18], followed by diastereospecific oxidative-rearrangement of the tetrahydro-β-carboline to a spiroxindole (Scheme 4). The most interesting feature of this methodology was that the presence or absence of the N_b_-benzyl protecting group permitted access into either the chitosenine series or the alstonisine series [30].

If Cook developed and studied the reaction condition for the “thermodynamic control”, the reaction conditions for “kinetic control” were developed by Bailey. An excellent *cis* stereocontrol was obtained, thus overcoming the need for unnatural d-tryptophan as the source of chirality [31,32,33,34]. The highest “kinetic control” was achieved with allyl esters of tryptophan and aryl aldehydes, very likely owing to a favorable π-stacking between the allyl/aryl groups, thus permitting the cyclization of the diaxial intermediate. As a consequence, only a small energetic difference between the axial and equatorial positions should be exist in the transition states of this reaction, if the modest stabilization through π-stacking might be sufficient to confer the *cis*-control. This hypothesis was also supported by the same *cis*-control observed in (*S*)-3-amino-4-(1*H*-indol-3-yl)butanenitrile reaction, in which a nitrile/aryl π-stacking is also possible [33]. Bailey’s conditions were also applied to the synthesis of many natural products [22,31,32,33,34,35].

**Scheme 5:**
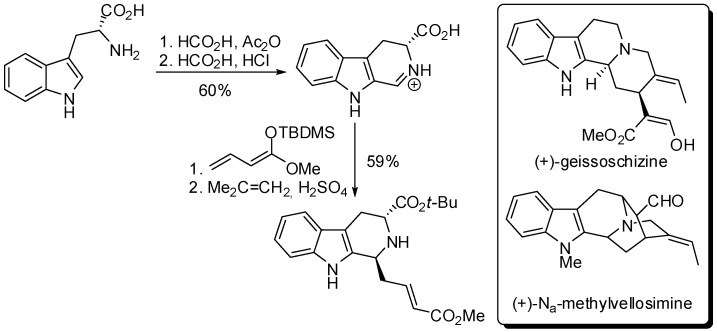
Key step in the synthesis of (+)-geissoschizine and (+)-*N*_a_-methylvellosimine.

It is worth mentioning also the concise asymmetric synthesis of the indole alkaloids (+)-geissoschizine and (+)-*N*_a_-methylvellosimine starting from d-tryptophan through a pseudo PSR. The synthesis involved the preparation of a dihydrocarboline iminium salt followed by a highly selective attack at C-3 of a vinyl ketene acetal from the face opposite to the carboxyl group at C-5 (Scheme 5) [36,37].

Very recently, Balalaie *et al.* used l-tryptophan propargyl ester and hydrazide in a Pictet–Spengler reaction in the presence of TFA, under “kinetic control”. The *cis*-tetrahydro-β-carboline propargyl esters were obtained as pure stereoisomers in 52%–73% yields (seven examples); whereas the carboline hydrazides were obtained in 65%–85% yields (five examples) as a mixture of *cis* and *trans* isomers with a ratio of 2:1 [38].

Shi and co-workers accomplished a high stereoselective Pictet–Spengler reaction of d-tryptophan methyl ester hydrochloride with piperonal and Cialis™ was thus efficiently synthesized in three steps in 82% overall yield [39]. The yield and *cis* stereoselectivity were solvent dependent and a *cis:trans* = 99:1 could be obtained when the reaction was performed in acetonitrile or nitromethane. The large difference of solubility of the diastereomers was accounted for the shift of the equilibrium between these two hydrochloride salts to the less soluble *cis* product.

**Scheme 6:**
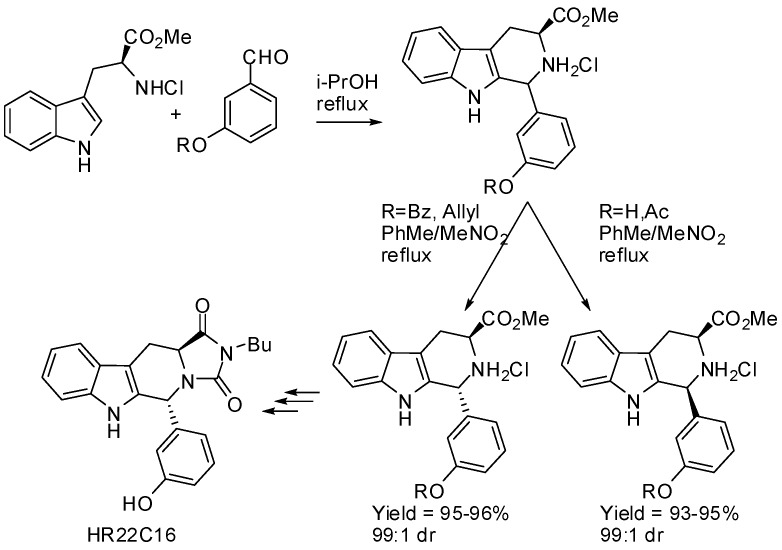
Stereoselective PSR by crystal-induced asymmetric transformation.

**Scheme 7:**
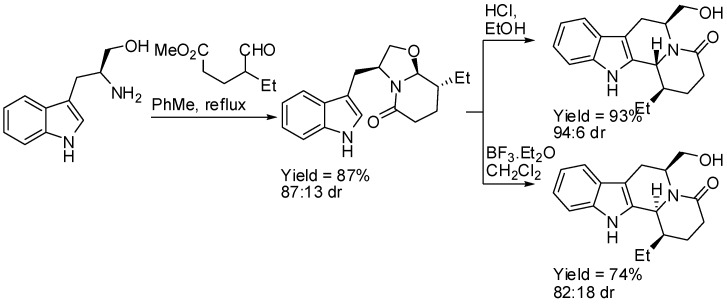
(*S*)-Tryptophanol in the enantioselective synthesis of indolo[2,3-*a*]quinolizidines.

Then, the reaction was extended to thirteen aldehydes, both aromatic and aliphatic, and high stereoselectivities and yields could be obtained, but the predominance of the *cis*- or *trans*-isomer was not predictable. It should be noted that yields were improved by performing the process in a modified two-step procedure: firstly the PSR was performed in isopropanol leading to epimeric mixtures and then the mixtures were submitted to a crystallization-induced asymmetric transformation of their hydrochloride salts [40]. The same research group applied this crystallization-induced asymmetric transformation to the stereoselective PSR of l-tryptophan methyl ester hydrochloride with 3-hydroxybenzaldehyde (Scheme 6). It is worth noting that unprotected or acetyl-protected aldehyde was transformed into the *cis-*derivative, whereas, benzoyl- or allyl-protected aldehyde afforded the *trans-*derivative, under the same conditions. Finally, HR22C16, a mitotic kinesin Eg5 inhibitor, could be readily synthesized from the enantiomerically pure (1*R*,3*S*)-1,3-disubstituted tetrahydro-β-carboline [41]. This procedure was also efficiently applied to the synthesis of a series of tetrahydro-β-carboline diketopiperazines as single isomers starting from l-tryptophan methyl ester hydrochloride and six aldehydes [42].

(*S*)-Tryptophanol was used as the starting material and as the source of chirality in the enantioselective two-step route to indolo[2,3-*a*]quinolizidines. After the stereoselective cyclo-condensation with methyl 4-ethyl-5-oxopentanoate, the resulting indole piperidone underwent intramolecular α-amidoalkylation on the indole 2-position under kinetic control, to give the 6,12b-*trans* indoloquinolizidine derivative (Scheme 7) [43]. It is worth noting that the use of BF·Et_2_O instead of HCl changed the stereoselectivity producing the 6,12b-*cis* isomer. Analogous results were obtained with dimethyl 3-(1-oxobutan-2-yl)pentanedioate.

**Scheme 8:**
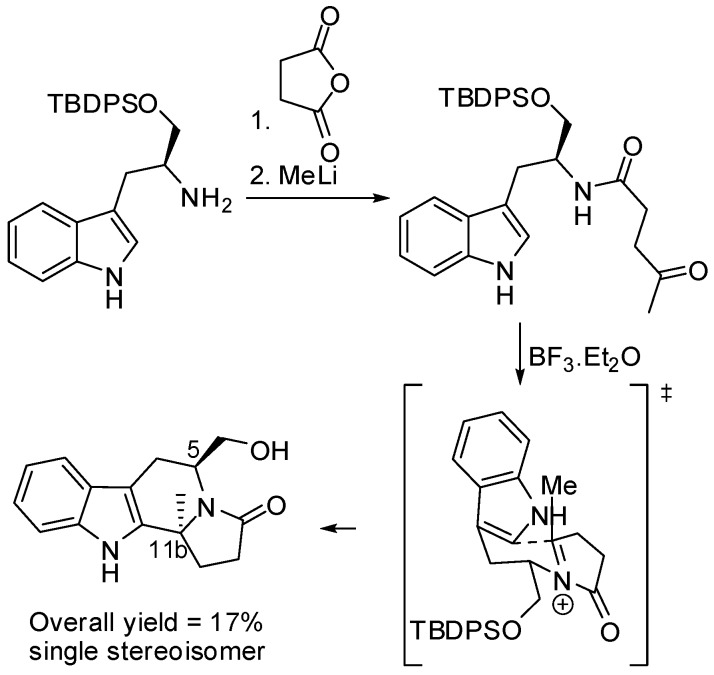
(*S*)-Tryptophanol in the enantioselective synthesis of indolo[2,3-*a*]indolizidinones.

The stereoselective synthesis of enantiomerically pure (5*S,*11b*S)*-indolo[2,3-*a*]indolizidinones was accomplished by a diastereoselective α-amidoalkylation reaction (Scheme 8). The substituent on carbon atom C-11b influenced the stereoselection, in fact no substitution on C-11b led to the *cis-*diastereomers [44].

Besides aldehydes, also ketones or oxazinanes/oxazolidines can be employed in the Pictet–Spengler reaction. For instance, cyclization of the imines prepared by the condensation of l-tryptophan methyl ester and aryl methyl ketones quantitatively furnished a mixture of (1*R*,3*S*) and (1*S*,3*S*)-1-aryl-3-isopropoxycarbonyl-1-methyl-1,2,3,4-tetrahydro-β-carbolines (1:1 to 5:1 dr) [45]. Again kinetic and thermodynamic controls governed the diastereoisomeric ratio being the (1*R,*3*S*)-diastereomer the most thermodynamically stable. Equilibration should occur under acidic conditions according to the pattern depicted in Scheme 3. Oxazinanes/oxazolidines were used as synthetic equivalents of aldehydes especially for aldehydes difficult to handle.

The stereochemical outcome was similar to that observed with simple aldehydes under thermodynamic control (72%–92% yields with 97:3 to 99:1 *trans:cis* ratio), while kinetic control is enhanced by sonication (68%–84% yields, up to 9:91 *trans:cis* ratio) [46].

## 3. Starting from Chiral Carbonyl Compounds

**Scheme 9:**
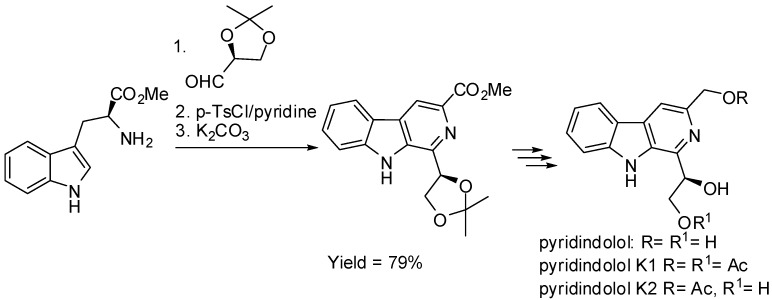
Total syntheses of (*R*)-pyridindolol, (*R*)-pyridindolol K1, and (*R*)-pyridindolol K2.

Besides derivatives of tryptophan, various chiral carbonyl compounds were used in order to induce enantioselectivity [47]. The total synthesis of (*R*)-pyridindolol, (*R*)-pyridindolol K1, and (*R*)-pyridindolol K2 (Scheme 9) in 66%, 41%, and 55% overall yield, respectively, was an example. In fact, although both the starting materials (l-tryptophan methyl ester and (*S*)-2,3-*O*-isopropylidene-glyceraldehyde) are chiral, the source of chirality in the products was the l-glyceraldehyde, because the chirality of tryptophan is lost during the aromatization of the six-membered nitrogen ring [48].

**Scheme 10:**
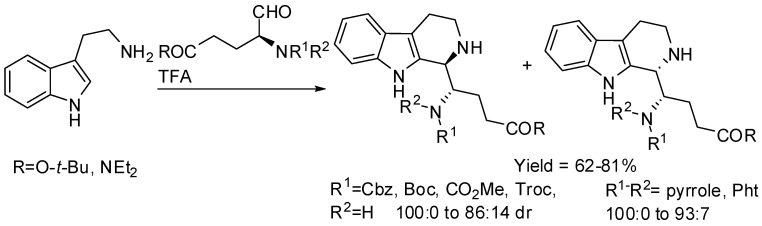
Diastereoselective PSR with chiral amino-aldehydes.

Tetrahydro-β-carbolines prepared from tryptamine and protected α-aminoaldehydes derived from l-glutamic acid were also studied (Scheme 10) [49]. The *cis*-diastereomer was mainly formed independently from the size of the protecting group with carbamates, in particular with Cbz group the *cis*-isomer is recovered as single diastereomer. On the other hand, pyrrole or phthalimide protecting groups overturned diastereoselectivity and, in particular, the *trans*-diastereomer was exclusively obtained from pyrrole-protected aminoaldehydes.

Later further studies on the PSR of d- and l-tryptophan with α-amino aldehydes derived from d- and l-amino acids showed that when both substrates have the same configuration (l,l or d,d, ‘mismatched’ situation), the *trans* diastereomer prevailed in the mixture of *cis* and *trans*-β-carbolines (65:35 to 100:0). In the ‘matched’ situation (l,d or d,l), only the *cis* diastereomer is recovered. A Felkin-Ahn model could explain this product distribution [50].

**Scheme 11:**
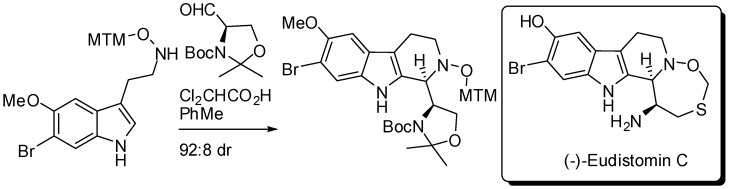
Diastereoselective PSR of a tryptamine derivative with Garner aldehyde.

During a stereocontrolled 18-step total synthesis of (−)-eudistomin C, Fukuyama and co-workers developed a Brønsted acid-catalyzed diastereoselective PSR of a tryptamine derivative with Garner aldehyde (Scheme 11) [51]. It is worth noting that with TFA the undesired diastereomer was the major product (3:1 dr), thus authors screened various conditions and found that the reaction afforded the desired diastereomer in the presence of a catalytic amount of dichloroacetic acid.

**Scheme 12:**
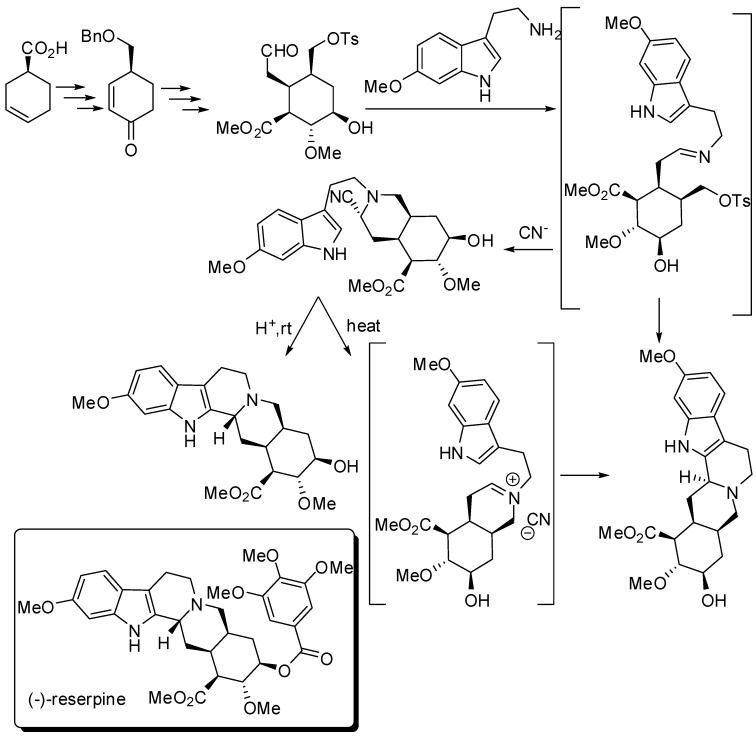
Key steps in the synthesis of (−)-reserpine.

Stork’s group prepared (−)-reserpine starting from (*S*)-3-cyclohexenecarboxylic acid, by modifying their racemic synthesis (Scheme 12) [52]. 

Interestingly, a reserpine-isoreserpine mixture is recovered if PSR occurred before closure of ring D, as well as if a tight iminium ion pair was formed before undergoing PSR. A “naked” iminium ion, instead, could assume the correct chair-axial conformation to lead to the correct C-3 stereochemistry.

Finally, an alternative route to the tetrahydro-β-carboline scaffold has been recently proposed with amino acid l-alanine or l-proline as the source of chirality. The synthesis contemplated the *de novo* construction of the indole ring with the appropriate substituents to give the PSR in the final step. A gram-scale total synthesis of (*S*)-eleagnine [53] and (*S*)-harmicine [54] in enantiopure form (>99% ee) was achieved.

## 4. Chiral Auxiliaries

The ideal chiral auxiliaries require ready availability, affordable cost, and, preferably, the accessibility of both enantiomers, as well as appropriate and efficient methods for their removal without affecting the optical integrity of the newly generated stereogenic center once the Pictet–Spengler reaction has been carried out, but these simple requirements seriously restrict the number of potentially useful candidates.

**Scheme 13:**
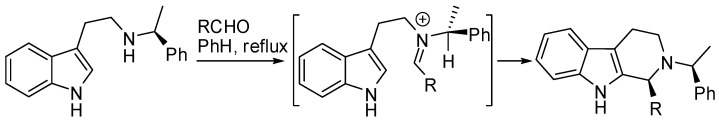
_-_α-Methylbenzylamine as chiral auxiliary.

The first attempt dates back to 1994 and used N_b_-α-methylbenzyl substituted 5,6-dimethoxy-tryptamine, which provided a 60:40 dr [55]. Then the reaction conditions were optimized and the best results (up to 86:14 and 69:31 dr for aromatic and aliphatic aldehydes, respectively) were obtained using trifluoroacetic acid in benzene at reflux (Scheme 13) [56]. Moreover, the auxiliaries benzyl- and 1-naphthyl-1-ethylamine provided similar diastereocontrol for aliphatic aldehydes (70:30 dr) [57].

**Scheme 14:**
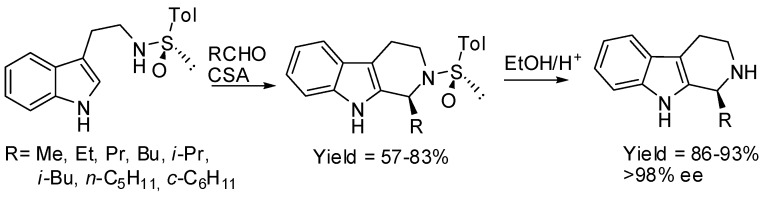
Chiral sulfoxide as chiral auxiliary.

**Scheme 15:**
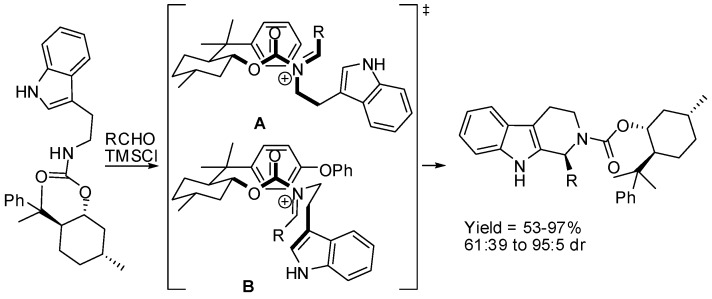
(−)-8-Phenylmenthylcarbamate as chiral auxiliary.

Chiral sulfoxides provided β-carbolines in 57%–63% yields and >98% ee (*S*-isomer) after removal of the auxiliary. It should be noted that camphorsulfonic acid was used as the acid catalyst. However, it did not influence stereochemistry, because either racemic or pure (+)-isomer gave the same diastereomeric ratio of the *N*-sulfinyl-β-carboline (Scheme 14) [58].

(*S*)-Tetrahydro-β-carbolines were also obtained from Pictet–Spengler reaction using a chiral carbamate of tryptamine (Scheme 15). In particular, (−)-8-phenylmenthylcarbamate was found superior to (−)-menthylcarbamate. Reaction conditions for auxiliary cleavage were not reported [59]. The transition state **A** was invoked to explain the stereoselectivity. If a 4-phenoxyphenyl substituent was present in the chiral auxiliary, the steric interactions favored the transition state **B** leading to (*R*)-isomers as the major product.

**Scheme 16:**
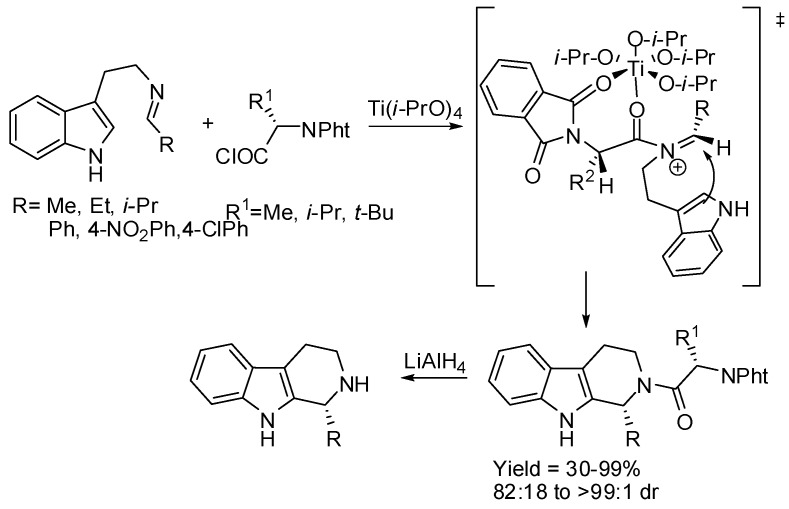
*N,N*-Phthaloyl-aminoacids as chiral auxiliaries.

*N,N*-phthaloyl-aminoacids could act as the chiral auxiliary when imines from tryptamine are treated with their chloride in the presence of titanium alkoxide. The reaction conditions were suitable for both aliphatic and aromatic aldehydes. However, removal of the auxiliary was obtained with LiAlH_4_ in only 66% yield (Scheme 16) [60]. The obtained carboline was *R*-configured that is enantiomeric with that obtained in the previous examples.

**Scheme 17:**
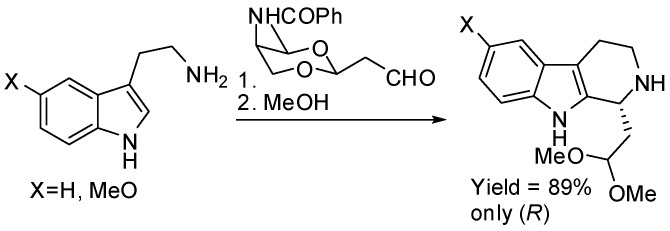
Malonaldehyde monocycloacetals as chiral auxiliary.

Tryptamine and 5-methoxytryptamine reacted with optically pure malonaldehyde monocycloacetals to give after Pictet–Spengler condensation and removal of the chiral auxiliary (*R*)-carbolines in 89% yield (Scheme 17) [61].

**Scheme 18:**
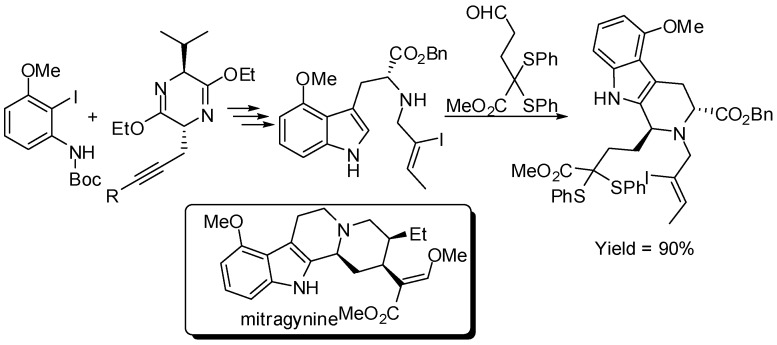
Synthesis and use of a tryptophan derivative in the synthesis of mitragynine.

A quite different use of chiral auxiliary was recently introduced by Cook research group [62,63,64]. They achieved benzo-substituted tryptophan derivatives by Larock indole synthesis of 2-iodo-N-Boc-anilines and propargyl-substituted Schöllkopf chiral auxiliary. Then the tryptophan derivative was used in PSR inside the synthesis of numerous natural products (Scheme 18 reports an example).

**Scheme 19:**
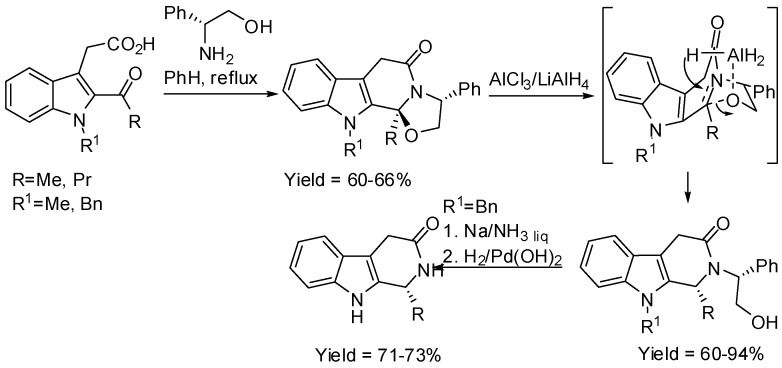
Cyclo-condensation of 2-acyl-3-indoleacetic acids with (*R*)-phenylglycinol.

Finally, another synthesis of β-carbolines is worth of mention, although it is not a proper Pictet–Spengler reaction. In fact, cyclo-condensation reactions between 2-acyl-3-indoleacetic acid derivatives and (*R*)-phenylglycinol provided tetracyclic lactams, which could easily be converted into enantioenriched 1-substituted tetrahydro-β-carboline alkaloids (Scheme 19) [65]. The most relevant feature of this reaction was the ring-opening reaction with retention of configuration under reductive conditions.

## 5. Preparing Chiral Compounds Beforehand

The previous sections reported classical chiral pool synthesis. All those reactions employed common chiral starting materials, the chirality of which is preserved in the remainder of the reaction sequence, or alternatively achiral compounds are transformed into chiral substrates by addition of easy removable auxiliary.

In the recent years, the development of asymmetric organocatalysis allowed the preparation of many structural different compounds, especially chiral carbonyl compounds. These compounds could find application in the efficient construction of enantioenriched tetrahydro-β-carbolines by PSR.

**Scheme 20:**
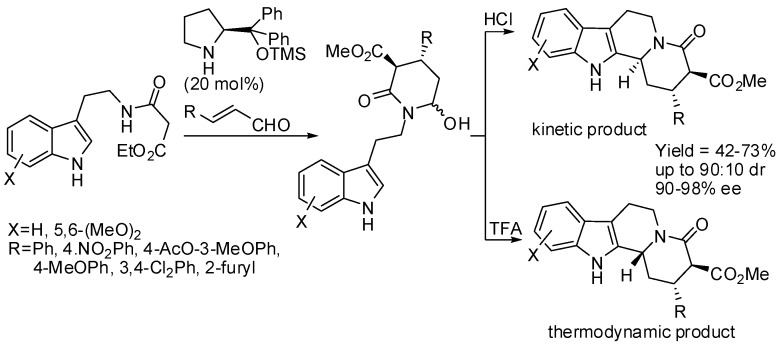
Enantioselective synthesis of indolo[2,3a]quinolizidines.

For instance, the organocatalytic conjugate addition of a nucleophile derived from tryptamine amide to cinnamaldehydes yielded a hemiaminal with two stereogenic centers (Scheme 20) [66,67]. The Pictet–Spengler cyclization was performed under both kinetic (hydrochloric acid at −78 °C) and thermodynamic control (trifluoroacetic acid at 70 °C) leading to the synthesis of alkaloid scaffolds. Unfortunately, β-alkyl-substituted acroleins decomposed in this reaction. It should be noted that, under thermodynamic control, diastereomer ratios depended on the reaction time and temperature, thus authors supposed that the α-epimer (kinetic product) was formed initially, and then it epimerized to the thermodynamically more-stable β-epimer.

This strategy was then applied to the synthesis of many indole alkaloids by the use of 5-hydroxypent-2-enal as the unsaturated aldehyde [68]. Depending on the reaction conditions, three out of four possible epimers of the corresponding quinolizidine alkaloids were enantio- and diastereoselectively prepared.

The group of Wu successfully attempted the reaction of Franzén with β-ketoamides instead of malono-monoamides, accessing to (2*R,*12b*S*)-indolo[2,3-*a*]quinolizidines in 56%–95% yields and 77%–98% ee (12 examples) [69]. Two points are worth noting: (i) conversely from the products obtained by Franzén, the C-2 was not asymmetric, because the enol form of the ketone was always recovered; (ii) pent-2-enal also worked in this reaction, although an inseparable 1:1 mixture of keto and enol form was recovered in 81% yield.

**Scheme 21:**
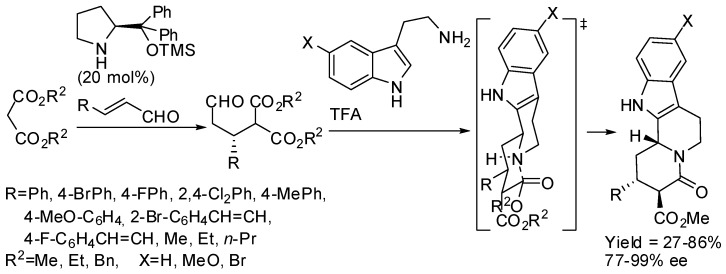
Syntheses of indoloquinolizidines by a Pictet-Spengler/lactamization cascade.

**Scheme 22:**
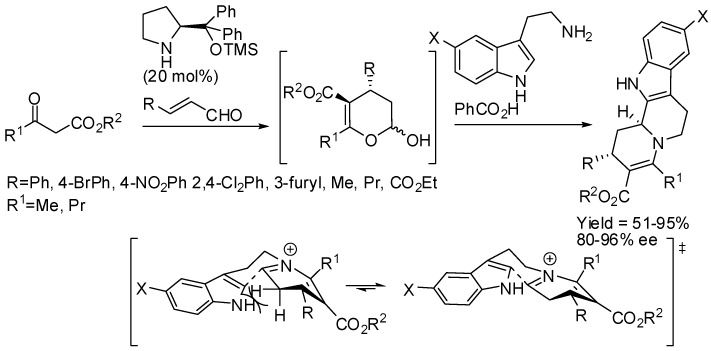
One-pot three-component Michael addition–Pictet–Spengler sequence.

The same research group then surmised that the coupling between malonate and α,β-unsaturated aldehydes could be performed before attaching malonate itself to tryptamine. Thus, they developed a diastereoselective cascade sequence for the preparation of *cis-*indolo[2,3-*a*]quinolizidines, in which Michael addition of malonate to α,β-unsaturated aldehydes was the first step, then PSR followed and lactamization was the final reaction step (Scheme 21). Also in this reaction, β-alkyl-substituted α,β-unsaturated aldehydes can be employed [70]. Then they extended the scope of this reaction to the synthesis of enantioenriched α-hemiaminal from α,β-unsaturated aldehydes and β-ketoesters, which in turn were cyclized with tryptamine in the presence of acid [71]. The stereochemistry of the products was defined in the Michael addition step and the diastereoselectivity of the PSR was shaped by the kinetically controlled reaction conditions (Scheme 22). Instead of classical Jørgensen-Hayashi catalyst, its 3,5-bis(trifluoromethyl) derivative was sometimes employed.

Rueping attempted this reaction with 1,3-cyclohexandiones [72]. The substrate scope was found to be general (20 examples) and optically active indoloquinolizidines were isolated as single diastereomers in 64%–85% yields with 78% to >99% ee. The absolute configuration of the indoloquinolizidines was determined by the X-ray analysis as (12b*S*,14*R*). The diastereoselectivity was attributed again to steric repulsion taking place between the incoming indole nucleophile and the protons of C-13.

In this Michael/Pictet–Spengler sequence, another explored substrate was α-oxo-γ-butyrolactam. The corresponding butyrolactam-fused indoloquinolizidines were recovered in 53%–87% yields with 75:25 to >95:5 dr and 90%–97% ee of the predominant (4*R*,5a*R*)-isomer (16 examples) [73]. Interestingly, the enantiomer (*i.e.*, that arising from unnatural proline) of the classical Jørgensen-Hayashi catalyst was employed. With regard to the mechanism, authors assumed a stereoselective *Re*-facial Michael addition of iminium ion to the enol of α-oxo-γ-butyrolactam. Then tautomerization and hydrolysis formed (4*S*)-2-hydroxy-4-alkyl-3,4,5,6-tetrahydropyrano[3,2-*c*]pyrrol-7(*2H*)-ones. This masked 1,5-dicarbonyl compound condensed with tryptamine to perform diastereoselective Pictet–Spengler reaction.

**Scheme 23:**
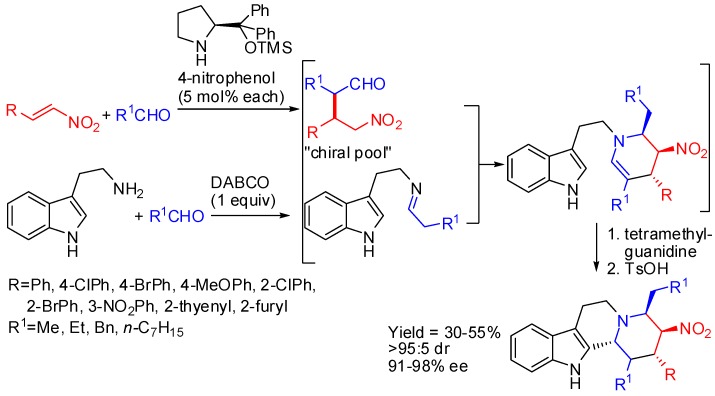
One-pot enantioselective construction of indoloquinolizidine derivatives with five contiguous stereocentres.

Another organocatalytic cascade one-pot reaction sequence has been developed for the construction of indoloquinolizidines. In this sequence the asymmetric Michael addition, catalyzed by Jørgensen-Hayashi catalyst, provided the chiral pool for the following aza-Henry reaction, hemiaminalization, dehydration, and PSR, providing indoloquinolizidines bearing five contiguous stereocentres (Scheme 23) [74]. It should be noted that only aliphatic aldehydes were used.

**Scheme 24:**
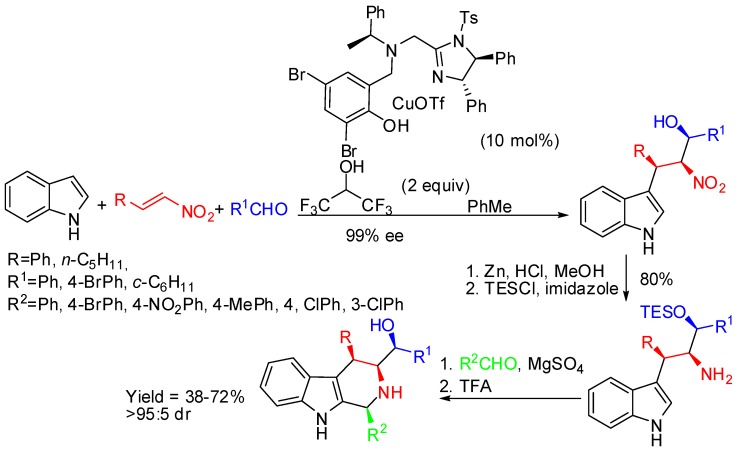
Four-step synthetic route to fully substituted chiral tetrahydro-β-carbolines.

Arai and co-workers set up a four-step synthetic route to fully substituted chiral tetrahydro-β-carbolines [75]. Enantioenriched (*R,S,S*)-tryptamine was obtained from three-component coupling of an indole, nitroalkene, and aldehyde catalyzed by imidazoline-aminophenol-CuOTf by a Friedel-Crafts/Henry cascade. Then reduction of-nitro group, protection of the hydroxy function allowed the PSR with aldehydes affording (1*S*,3*S*,4*R*)-tetrahydro-β-carbolines (Scheme 24).

Jia *et al.* reported and example of PSR into their report on the enantioselective Friedel–Crafts alkylation of indoles with β-CF_3_-β-disubstituted nitroalkenes. Actually, (*R*)-3-(1,1,1-trifluoro­3-nitro-2-phenylpropan-2-yl)-*1H*-indole (96% ee) was reduced and then cyclized with benzaldehyde under TFA catalysis, in 78% yield, but only a 67:33 diastereomeric ratio was achieved although the enantiomeric excess of both isomers reflected that of the starting material [76].

**Scheme 25:**
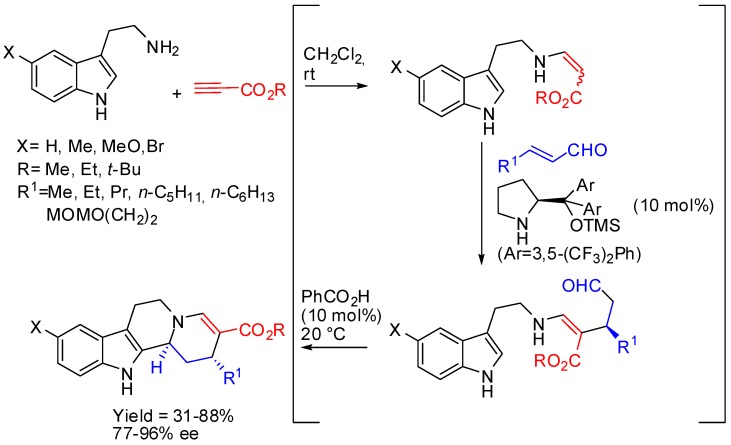
One-pot three-component syntheses of indoloquinolizidines.

The asymmetric organocatalyzed one-pot three-component cascade reaction of tryptamines, alkyl propiolates, and α,β-unsaturated aldehydes represented another instance, in which a chiral substrate was prepared before PSR. The reaction could be carried out in a one-pot sequence leading to the (2*R*,12b*S*)-stereoisomer (Scheme 25) [77].

**Scheme 26:**
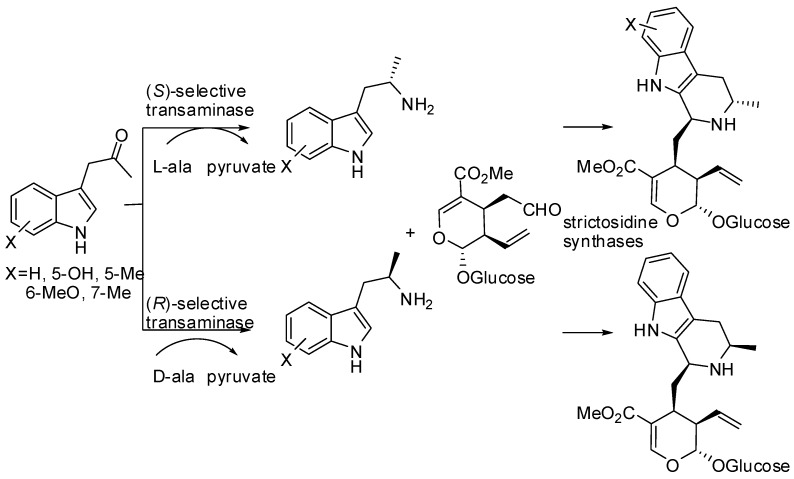
Stereoselective synthesis of C3-methylated strictosidine derivatives.

Finally, an enzymatic synthesis of optically pure C-3 methyl-substituted strictosidine derivatives, key intermediates in the biosynthesis of several pharmaceutically relevant monoterpenoid indole alkaloids, was performed. Both enantiomers of methyl tryptamine were prepared by either (*R*)-selective transaminase from *Arthrobacter* species or (*S*)-selective transaminase from *Silibacter pomeroyi* or *Bacillus megaterium*, thus providing the first stereogenic center at the β-carboline core with up to >98% enantiomeric excess. The chiral tryptamines were then condensed with secologanin in a diastereoselective Pictet–Spengler reaction catalyzed by strictosidine synthase from *Ophiorriza pumila* or *Rauvofolia serpentina* (Scheme 26) [78]. Strictosidine synthase clearly controlled the diastereoselectivity, in fact both enantiomers at C-3 of the amine led to an (*S*)-configured center at C-1 of the carboline. Finally the reaction can be carried out either in a one-pot cascade or in a stepwise fashion in up to 97% yield.

## 6. Conclusions

The discovery by Pictet and Spengler provided chemists with a powerful method for the synthesis of natural biologically active compounds, especially in the total synthesis of alkaloids. Over 100 years since its discovery and until today, there has always been high demand for the development of new efficient catalytic systems Impressive results have been obtained with the asymmetric Pictet–Spengler reaction starting from chiral tryptamine and carbonyl compounds derivatives. In the last years, however, the classical “chiral pool” methodology, which starts from naturally occurring compounds, has been replaced by asymmetric synthesis from achiral starting materials and chiral acids or hydrogen-bond donors. Cascade multi-component reactions resemble the classical “chiral pool” methodology, since they allow the preparation of enantioenriched compounds, before Pictet–Spengler cyclization. On the other hand a large number of modern syntheses allow the creation of the asymmetric carbons in a single-step reaction.

## Abbreviations

The following abbreviations are used in this manuscript:

Ac	Acetyl
Boc	*tert*-Butoxycarbonyl
Bz	Benzoyl
Cbz	Benzyloxycarbonyl
CSA	Camphorsulfonic acid
DABCO	1,8-Diazabicyclooctane
MTM	Methylthiomethyl
Pht	Phthalimide
TBDMS	*tert*-Butyldimethylsilyl
TBDPS	*tert*-Butyldiphenylsilyl
TES	Triethylsilyl
TFA	Trifluoroacetic acid
Tol	4-Methylphenyl (Tolyl)
Troc	2,2,2-Trichloroethoxycarbonyl
Ts	Methylphenylsulfonyl (Tosyl)
